# Epidemiology, genetic variants and clinical course of natural infections with *Anaplasma phagocytophilum* in a dairy cattle herd

**DOI:** 10.1186/s13071-017-2570-1

**Published:** 2018-01-08

**Authors:** Cornelia Silaghi, Marion Nieder, Carola Sauter-Louis, Gabriela Knubben-Schweizer, Kurt Pfister, Martin Pfeffer

**Affiliations:** 1 Comparative Tropical Medicine and Parasitology, Faculty of Veterinary Medicine, Ludwig-Maximilians-University Munich, Munich, Germany; 2 Present Address: Federal Research Institute for Animal Health, Institute of Infectology, Friedrich-Loeffler-Institute, Riems, Germany; 3 Institute for Animal Hygiene and Veterinary Public Health, Faculty of Veterinary Medicine, University of Leipzig, Leipzig, Germany; 4 Clinic for Ruminants with Ambulatory and Herd Health Services at the Centre for Clinical Veterinary Medicine, Ludwig-Maximilians-University Munich, Munich, Germany; 5 Federal Research Institute for Animal Health, Institute of Epidemiology, Friedrich-Loeffler-Institut, Riems, Germany

**Keywords:** *Anaplasma phagocytophilum*, Cattle, Tick-borne fever, *Ixodes ricinus*, *16S rRNA* gene, *groEL* gene, *msp2* gene, *msp4* gene, Germany

## Abstract

**Background:**

*Anaplasma phagocytophilum* is an obligate intracellular, tick-transmitted bacterium that causes granulocytic anaplasmosis in humans and several mammalian species including domestic ruminants where it is called tick-borne fever (TBF). Different genetic variants exist but their impact with regard to putative differences in host associations and pathogenicity are not yet completely understood.

**Methods:**

Natural infections with *A. phagocytophilum* in a dairy cattle herd in Germany were investigated over one pasture season by using serology, haematology, blood chemistry and polymerase chain reaction (PCR). Sequence analysis of partial *16S rRNA*, *groEL*, *msp2* and *msp4* genes of *A. phagocytophilum* was carried out in order to trace possible genetic variants and their relations between cattle, roe deer (*Capreolus capreolus)* and ticks (*Ixodes ricinus*) in this area.

**Results:**

In total 533 samples from 58 cattle, 310 ticks, three roe deer and one wild boar were examined. Our results show (i) typical clinical symptoms of TBF in first-time infected heifers, such as high fever, reduced milk yield, lower limb oedema and typical haematological and biochemical findings such as severe leukopenia, erythropenia, neutropenia, lymphocytopenia, monocytopenia, a significant increase in creatinine and bilirubin and a significant decrease in serum albumin, γ-GT, GLDH, magnesium and calcium; (ii) a high overall prevalence of *A. phagocytophilum* infections in this herd as 78.9% (15/19) of the naïve heifers were real-time PCR-positive and 75.9% (44/58) of the entire herd seroconverted; and (iii) a high level of sequence variation in the analysed genes with five variants of the *16S rRNA* gene, two variants of the *groEL* gene, three variants of the *msp2* gene and four variants in the *msp4* gene with certain combinations of these variants.

**Conclusions:**

In cattle particular combinations of the genetic variants of *A. phagocytophilum* occurred, whereas three roe deer showed different variants altogether. This is indicative for a sympatric circulation of variants in this small geographical region (< 1 km^2^). Both re- and superinfections with *A. phagocytophilum* were observed in five cattle showing that infection does not result in sterile immunity. For prevention of clinical cases we suggest pasturing of young, not pregnant heifers to reduce economical losses.

**Electronic supplementary material:**

The online version of this article (10.1186/s13071-017-2570-1) contains supplementary material, which is available to authorized users.

## Background

The tick-transmitted obligate intracellular, gram-negative bacterium *Anaplasma phagocytophilum* occurs in intracytoplasmatic vacuoles in neutrophilic and eosinophilic granulocytes of infected mammalian hosts [1]. In Europe, the main vector is the hard tick *Ixodes ricinus* and the main reservoir hosts discussed are roe deer (*Capreolus capreolus*) and other wildlife ruminants, but also wild boars (*Sus scrofa*), hedgehogs (Erinaceinae) and other small mammal species [2]. *Anaplasma phagocytophilum* causes granulocytic anaplasmosis in humans, horses, dogs and cats and tick-borne fever (TBF) in ruminants [3]. Typical clinical signs of TBF include fever, sudden decrease in milk production, inappetence, lethargy, lower limb oedema; typical laboratory observations are leukopenia and thrombocytopenia [4–7]. TBF causes economical losses due to the drastic decrease in milk production and is considered to be underdiagnosed in cattle [4, 6, 8]. Only few reports on natural infections on herd basis exist. They all describe two peaks of clinical cases in spring and autumn, matching the highest activity levels of *I. ricinus* ticks [6, 9–13]. A high genetic variation of *A. phagocytophilum* was described previously for several partial gene fragments [14]. Different *A. phagocytophilum* strains seem to have varying infectivity for different mammalian species [1]. Multilocus sequence typing (MLST) showed that the population structure of *A. phagocytophilum* might be semiclonal with a uniform clonal complex 1 with strains from humans, dogs and horses and a higher heterogeneity in clonal complex 2 with strains from wild and domestic ruminants [2]. In Germany, the first laboratory confirmed case of TBF occurred in 2010 in a dairy cattle herd in North-Rhine-Westphalia [8]. This study presents the follow-up diagnosis in the same herd. The objectives were to identify natural infections with *A. phagocytophilum* in a dairy cattle herd by cytology, serology, haematology, blood chemistry and polymerase chain reaction (PCR) as well as genetic variants of the partial *16S rRNA*, *groEL* (heat-shock protein HSP60, also known as caperonin 60), *msp2* and *msp4* (major surface proteins 2 and 4) genes. The results will allow to determine associations between cattle, wild animals and ticks in the area under investigation and to identify effective control measures.

## Methods

### Dairy cattle herd

This study was performed from April 2011 to February 2012 in a dairy cattle herd in North-Rhine-Westphalia, Germany, where tick-borne fever is endemic [8]. The minimal stock was 39 cows and 11 heifers (in this paper heifer is used synonymous for first calf heifer) in May 2011 and the maximal stock was 39 cows and 19 heifers in July 2011. The animals were cross-breeds of Red and Black Holstein Friesian and German Simmental in a closed breeding system. The herd went to pasture from May 9th until October 27th during daytime hours between milking times and stayed in a freestall barn overnight and during the winter months. Cows were pastured in turns on four different 2.5 ha to 4.0 ha pastures from 250 m to 400 m above sea level. Pastures were surrounded by small forests, contained watering places and were often frequented by wild animals like roe deer and wild boars, but not by red deer (*Cervus elaphus*). Heifers went to the pasture for the first time, whereas the cows had been to the pastures for one or more pasture periods before. Tick infestation on the animals was regularly observed during milking times. Eight of the 19 heifers (nos. 7, 15, 16, 23, 28, 52, 53, 61) were treated by the farmer with repellents (flumethrin: Bayticol® Pour-on, 10 mg/ml, Bayer AG, Germany) according to the product information every 3 weeks from May 15th until they calved. Effectiveness against ticks is stated with 3 weeks, the withholding period on meat is 5 days and on milk 8 days. Therefore, treatment of heifers was stopped when they calved and lactating cows had not been treated with repellents at all.

### Clinical examination and blood sampling

**Fig. 1:**
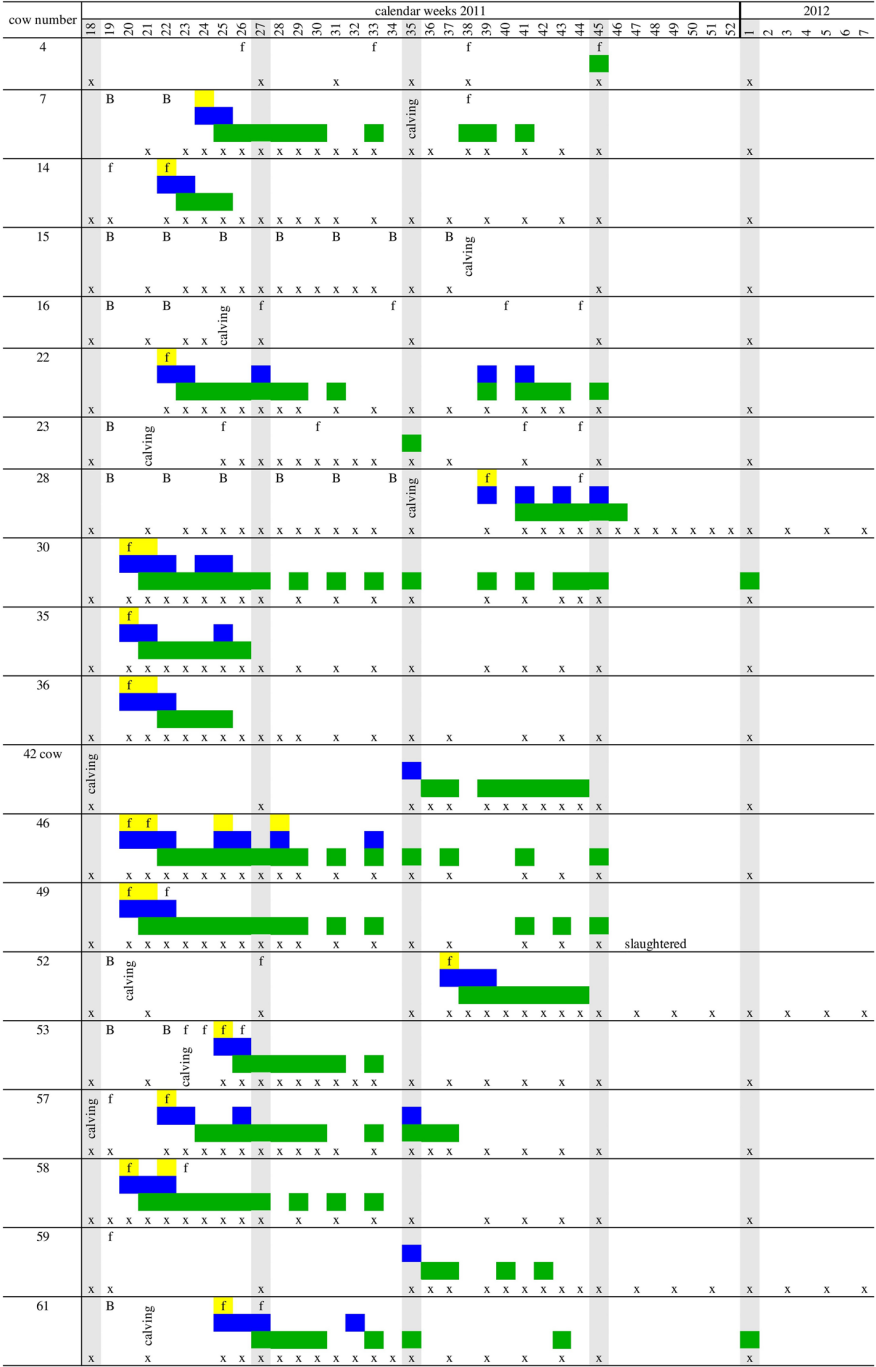
Course of infection of observed heifers and cows in the herd in relation to clinical, microscopial and serological findings. Yellow, morulae in buffy coat smears; blue, real-time PCR-positive for *A. phagocytophilum*; green, positive titer >1:100; grey, herd screening; f, fever; B, Bayticol®-treatment; x, sampling point

Blood samples of the herd were taken prior to pasturing in May 2011 and then every other month until January 2012 (“herd screening”). Cytological, serological and PCR examinations were performed as described below. Previous cases of TBF in the herd showed that clinical infections with *A. phagocytophilum* become most obvious in lactating naïve heifers [8]. Therefore, rectal body temperature was measured daily in heifers and a body temperature ≥39.5 °C was considered suspicious for an infection. Subsequently, these heifers were observed by the farmer for reduced milk yield, discharge from eyes and nose, lower limb oedema and stiff walking. Blood was taken for detection of *A. phagocytophilum* and in case of positive PCR results, additional blood samples were taken weekly for the following 6–8 weeks and thereafter every other week for further 6–8 weeks (Fig. 1). All blood samples for diagnostic purposes were taken from tail veins into EDTA- and serum-tubes (S-Monovette, 10 ml, Sarstedt AG & Co, Nümbrecht, Germany).

### Cytological, serological, haematological and biochemical examination

Buffy coat smears were prepared from every sample and stained with Giemsa for microscopical investigations for morulae of *A. phagocytophilum.* Serum samples were analysed for *A. phagocytophilum* antibodies by indirect immunofluorescence (IFAT) with the MegaScreen® Fluoanaplasma ph. slides (MegaCor, Hörbranz, Austria) and anti-bovine IgG-conjugate in a dilution of 1:80 (Sigma-Aldrich, Taufkirchen, Germany). A serum titer starting from 1:100 was considered positive whereas 1:50 was considered as borderline titer. Blood of *A. phagocytophilum-*positive heifers was also examined for haematological and biochemical parameters. EDTA-blood was used for determination of leukocytes, erythrocytes, thrombocytes, hematocrit, haemoglobin and a differential blood count. Urea nitrogen, creatinine, total protein, albumin, total bilirubin, phosphor, magnesium, calcium, sodium, potassium, chloride and the activities of aspartate aminotransferase (AST), γ-glutamyltransferase (γ-GT), glutamate dehydrogenase (GLDH) and creatine kinase (CK) was measured in the serum. The examinations were performed according to the laboratory’s standard (Clinic for Ruminants with Ambulatory and Herd Health Services, Faculty of Veterinary Medicine, Ludwig-Maximilians-University Munich). For the statistical analysis (see below) of the blood parameters samples from day 0 (day of clinical signs in combination with first real-time PCR positive result), day 7, day 14, day 21 and day 28 were compared with samples from day 200 after first real-time PCR positive result.

### Environmental investigations

For detection of prevalence and different genetic variants of *A. phagocytophilum* in the pasture area, ticks and professionally hunted wild animals were examined as follows: Ticks were collected on 5 days from April to June 2011 on four pastures in areas containing bushes and trees by the flagging method. Additionally, engorged ticks were collected from cows during milking time and from professionally hunted game animals from the pasture area in May and June 2011. Ticks were stored in 70% ethanol for individual identification to species level under a stereomicroscope [15] and were separated by sex, stage and date of collection. For DNA-extraction and PCR all adults were examined separately whereas nymphs and larvae were pooled with a maximum of five individuals in a tube. Spleen tissue samples from game animals were taken with a sterile punch and stored in 70% ethanol for further analysis.

### DNA extraction and real-time PCR

DNA extraction of blood and spleen samples was accomplished with Maxwell® 16 LEV Blood DNA Kit (Promega, Madison, USA) according to the manufacturer’s instructions in Maxwell® 16 MDx (Promega, Madison, USA). DNA extraction of ticks was performed with QIAmp DNA Mini Kit (Quiagen, Hilden, Germany) according to the manufacturer’s instructions with modifications. Ticks were macerated individually with 0.6 g ceramic beads with 1.4 mm diameter (peqLab) and 100 μl PBS-Buffer at 5000× *rpm* for 5 min in a tissue homogenizer (Precellys®24, Bertin technologies, Montigny-le-Bretonneux, France), kept overnight at 56 °C with 100 μl ATL-Buffer and 20 μl Proteinase K. Final eluation was done with 50 μl AE-Buffer. Extracted DNA was tested for quality and quantity with a spectrophotometer (NanoDrop® ND-1000, PeqLab, Erlangen, Germany). Blood and spleen samples were screened for *A. phagocytophilum* with real-time PCR targeting the *msp2* gene [16, 17]. All samples were tested in duplicates along with positive and negative controls (see Additional file 1: Table S1 for all cycling conditions and primers used).

### PCR genotyping and sequence analysis

PCR genotyping and sequence analysis was performed for the first real-time PCR-positive sample of a cow or a heifer and for positive samples of roe deer and ticks. To detect possible new infections with different genetic variants, further samples from certain positive heifers were additionally tested. Investigations included a nested-PCR targeting a 497 bp part of the *16S rRNA* gene of *A. phagocytophilum* [18], a heminested PCR assay for a 530 bp part of the *groEL* gene [19], a conventional PCR targeting a 893 bp part of the *msp2* gene [20] and a nested PCR of a 350 bp *msp4* gene [21, 22]. PCRs were performed according to Silaghi et al. [23] (Additional file 1: Table S1). Amplicons were visualized using UV light after staining with GelRed® (Biotium, Hayward, USA) and 2.0% agarose gel electrophoresis and purified with QIAquick PCR Purification Kit according to the manufacturer’s instructions (Qiagen, Hilden, Germany). Sequencing with forward and reverse primers of the nested reactions was performed by Eurofins MWG Operon (Martinsried, Germany). The obtained sequences were analysed with: Chromas©Lite (http://technelysium.com.au), BLASTn (http://blast.ncbi.nlm.nih.gov/Blast.cgi under “nucleotide blast”), Reverse Complement (http://www.bioinformatics.org/sms/rev_comp.html) and ClustalW2 (http://www.ebi.ac.uk/Tools/msa/clustalw2/) [24].

### Statistical analysis

Data were examined visually for normal distribution using box-and-whisker plots. The calculation of means, medians and standard deviations (SDs) was done using SPSS (IBM, version 21). Concentrations of different blood parameters were compared using Wilcoxon signed-rank test for paired samples, whereby the concentrations of different days during the period of infection were compared with the concentrations at day 200, which was outside the period of infection. A *P*-value of less than 0.01 was considered statistically significant.

## Results

In total 15 out of the 19 heifers (78.9%) and one out of the 39 cows (2.6%) were positive in real-time PCR for *A. phagocytophilum* during the pasture period. This corresponds to an overall prevalence of 27.6% for the whole herd.

### Clinical picture

All *A. phagocytophilum*-positive heifers except for two showed typical clinical symptoms of TBF at first infection (Table 1). One cow (no. 42) and one heifer (no. 7) were not examined for clinical symptoms before their positive results and one heifer (no. 59) showed no clinical symptoms at all (Fig. 1). Six heifers developed clinical symptoms after 8–13 days of first exposure to the tick-infested area in May, five heifers after 21–44 days in June. Only two heifers (nos. 28 and 52) developed TBF in autumn and one of them was brought for the first time to the pasture in September. Thirteen out of the 16 animals that were real-time PCR-positive for *A. phagocytophilum* had one to 5 days fever before their first real-time PCR positive result. The mean rectal body temperature in the period of fever was 40.5 °C (range: 39.5–41.7 °C). All 13 heifers showed a sudden decrease in milk production, nine had discharge from eyes and nose and five showed lower limb oedema and stiff walking (Table 1). Fourteen out of the 16 real-time PCR-positive animals had more than one sample with a positive real-time PCR result (Fig. 1). Seven heifers (nos. 22, 28, 30, 35, 46, 57, 61) had positive samples after at least 1 week of a negative result and they showed no clinical signs at this time. All affected animals recovered without antibiotic treatment after an average duration of 1 week.

**Table 1:** Analysed sequences of *A. phagocytophilum* in this herd in combination with clinical symptoms of the infected animals

Cow no./ tick no./roe deer no.	Date of positive PCR	Ct-value	Clinic	*16S rRNA*	*groEL*	*msp2*	*msp4*
7	19.06.11	23	not examined	**16S-20 (W)**	**g-18 (X)**	**M2-26**	**M4-49**
14	04.06.11	17	F, RM	**16S-20 (W)**	**g-18 (X)**	**M2-26**	**M4-49**
22	30.05.11	15	F, RM, LLO	**16S-20 (W)**	**g-18 (X)**	**M2-26**	**M4-49**
	09.07.11	28.5	no clinic	16S-20 (W)	too short	negative	M4-50
	03.10.11	28	no clinic	16S-20 (W)	g-15 (N)	negative	M4-51
28	03.10.11	20	F, RM, DC	16S-20 (W)	g-15 (N)	negative	M4-51
30	17.05.11	27.5	F, RM, DC	16S-20 (W)	g-18 (X)	too short	M4-50
35	17.05.11	15	F, RM	**16S-20 (W)**	**g-18 (X)**	**M2-26**	**M4-49**
36	19.05.11	13.5	F, RM, DC	16S-20 (W)	too short	M2-26	M4-49
42	04.09.11	36	not examined	16S-20 (W)	too short	negative	M4-49
46	22.05.11	19.5	F, RM, DC, LLO	**16S-20 (W)**	**g-18 (X)**	**M2-26**	**M4-49**
	21.08.11	30	no clinic	negative	negative	negative	M4-50
49	19.05.11	31.5	F, RM, DC, LLO	16S-20 (W)	g-15 (N)	negative	M4-51
52	14.09.11	19.5	F, RM, LLO	16S-20 (W)	too short	M2-27	M4-51
53	22.06.11	14	F, RM, DC	16S-20 (W)	g-18 (X)	negative	M4-49
57	04.06.11	18.5	F, RM, DC	**16S-20 (W)**	**g-18 (X)**	**M2-26**	**M4-49**
	04.09.11	30	no clinic	16S-20 (W)	negative	negative	M4-49
58	17.05.11	24.5	F, RM, DC	**16S-20 (W)**	**g-18 (X)**	**M2-26**	**M4-49**
	09.07.11	31	no clinic	16S-20 (W)	negative	negative	negative
59	04.09.11	26.5	no clinic	16S-22 (Y)	too short	negative	M4-13 (*n*)
61	22.06.11	18	F, RM, DC, LLO	**16S-20 (W)**	**g-18 (X)**	**M2-26**	**M4-49**
	09.07.11	22	no clinic	16S-20 (W)	negative	M2-27	M4-51
Tick 83^a^	13.05.11	29		16S-7 (I)	negative	negative	negative
Tick 90^b^	06.06.11	20		16S-20 (W)	too short	negative	M4-51
Tick 94^b^	06.06.11	23		16S-20 (W)	too short	negative	M4-49
Tick 96^b^	06.06.11	19		16S-20 (W)	too short	negative	M4-51
Tick 167^a^	25.06.11	30		16S-22 (Y)	too short	negative	M4-13 (*n*)
Roe deer 1	26.06.11	30		16S-21 (X)	negative	M2-9 (J)	too short
Roe deer 2	17.09.11	27.5		16S-21 (X)	negative	negative	negative
Roe deer 3	15.10.11	30		16S-19 (V)-like sequence	too short	negative	too short

*Abbreviations*: *F* fever, *RM* reduced milk yield, *DC* discharge from eyes and nose, *LLO* lower limb oedema and stiff walking

*Note*: Nomenclature of gene variants is following previous denominations [17, 23, 25] with numbers after the gene abbreviation. The letter codes shown for some of these variants are an alternative, also unofficial nomenclature. Particular combinations of these four gene variants were frequently observed with 16S-20 (W), g-18 (X), M2-26, and M4-49 are given in bold; negative = gene locus could not be amplified; too short = gene locus was amplified but the sequence read was too short for full comparison and thus for allocation to a particular variant. Ct-values are provided as mean of two independent real-time PCRs targeting the *msp*2-gene, which was used for screening. Please note that tick screening was done only once. A ct-value ≤38 was considered positive for *A. phagocytophilum* DNA

^a^Collected from roe deer

^b^Collected from heifer

### Cytological, serological, haematological and biochemical examination

In total, 533 EDTA and 533 serum samples were taken. Morulae could be observed in leukocytes in every first real-time PCR-positive sample of the heifers except for the two animals that where found positive in the herd screening (nos. 42, 59, see above). They could be seen in the first sample (*n* = 14) on the day of fever or also in the second sample (*n* = 4) 1 week later. Morulae were always found in samples of heifers with clinical symptoms and positive real-time PCR. In real-time PCR-positive samples, when the heifers did not show any clinical signs, morulae could only be observed in two heifers (nos. 46, 58) (Fig. 1). In total 75.9% of the herd (44/58 animals) showed antibodies over the whole pasture season and 44.8% (26/58 animals) had positive titers of ≥1:100 (Table 2). Seroprevalence with titers of ≥1:100 was 0.0% prior to pasturing in May 2011 and increased to a maximum of 36.2% in July. In September seroprevalence decreased to 27.3%, in November to 17.9% and was 3.8% in January 2012 (Additional file 2: Figure S1). The highest titers (up to 1:6400) occurred in July 2011, parallel to the seroprevalence peak (Table 2, Additional file 2: Figure S1). Antibodies were always observed 1 week after the first PCR-positive result for *A. phagocytophilum* and lasted from 2 weeks (nos. 42, 59) up to nine consecutive weeks (no. 49) (Fig. 1). Some animals (nos. 30, 49, 58) may have even longer lasting antibody titres, but as we shifted the sampling intervals to 2 weeks, we cannot rule out a re- or superinfection in between. (Fig. 1). In haematological and biochemical examinations (Table 3) we found a significant leukopenia, erythropenia, neutropenia (only segmented neutrophils), lymphocytopenia and monocytopenia in animals with positive real-time PCR results (day 0) *versus* animals with negative real-time PCR results (day 200). We also found a significant increase in the parameters creatinine and bilirubin and a significant decrease in the parameters albumin, γ-GT, GLDH, magnesium and calcium between animals at these time points (Table 3).

**Table 2:** Serum titers of IgG against *A. phagocytophilum* in a dairy cattle herd (heifers and cows) from May 2011 to January 2012

Month	Total no. of cows^a^	Titers								
		0	1:50	1:100	1:200	1:400	1:800	1:1600	1:3200	1:6400
May	50 (39, 11)	48 (37, 11)	2 (2, 0)	0	0	0	0	0	0	0
July	58 (39, 19)	29 (20, 9)	8 (7, 1)	7 (5, 1)	8 (3, 5)	1 (0, 1)	0	2 (1, 1)	2 (2, 0)	1 (1, 0)
September	55 (36, 19)	30 (19, 11)	10 (7, 3)	11 (7, 4)	3 (3, 0)	1 (0, 1)	0	0	0	0
November	56 (37, 19)	33 (21, 12)	13 (8, 5)	7 (3, 4)	0	0	3 (2, 1)	0	0	0
January	52 (34, 18)	40 (25, 15)	10 (9, 1)	1 (0, 1)	1 (0, 1)	0	0	0	0	0

^a^Numbers of cows and heifers, in that order, are indicated in parentheses

**Table 3:** Haematological and biochemical findings for 10 heifers after first real-time PCR positive result for *A. phagocytophilum*

Parameter	Reference values	Days after first positive real-time PCR result					
		0	7	14	21	28	200
Leukocytes	4–10 G/l	3.55 (1.9–6.2)^*^	5.8 (2.9–9.8)	6.95 (3.3–10.6)	6.85 (2.9–19.5)	6.95 (4.0–11.3)	8.4 (6.5–14.4)
Erythrocytes	5–8 T/l	6.16 (4.8–9.1)^*^	6.12 (4.9–8.3)	6.495 (5.3–9.3)^*^	6.135 (4.9–8.6)^*^	6.42 (5.0–9.2)	9.51 (6.3–11.6)
Haemoglobin	10–13 g/dl	9.75 (7–15.1)	9.3 (7.4–14.8)	10.25 (8.2–14.5)^*^	9.35 (8.3–14.3)^*^	9.75 (7.8–15.1)	14.4 (9.4–17.6)
Haematocrit	30–36%	29.8 (22.3–49.3)	29.7 (24.9–45.9)	33.05 (26.5–47.5)	30.2 (26.5–46.6)	31.4 (25.4–48.4)	40.6 (29.9–49.7)
Thrombocytes	200–800 G/L	87 (22–333)	192 (100–436)	276.5 (61–584)	312 (96–513)^*^	338.5 (28–484)	175 (94–421)
Banded neutrophils	0–0.1 G/l	0.24 (0–0.6)	0.23 (0.1–1.8)	0.405 (0.07–1.04)	0.165 (0.04–5.66)	0.445 (0.05–1)	0.245 (0–1.68)
Seg. neutrophils	0.85–1.53 G/l	1.52 (0.9–3.8)	1.98 (0.7–3.5)	2.555 (0.8–5.15)	2.435 (1.3–9.6)	3.615 (1.1–5.5)	4.18 (3.1–8.4)
Lymphocytes	2.5–5.5 G/l	1.09 (0.6–2.4)^*^	3.2 (0.9–5.2)	3.62 (0.91–5.1)	3.025 (1.16–6.79)	3.705 (1.08–6.14)	2.46 (1.56–4.3)
Monocytes	0–0.2 G/l	0.02 (0–0.32)^*^	0.17 (0–0.69)	0.225 (0.03–0.9)	0.205 (0–0.47)	0.24 (0.04–0.77)	0.2 (0.07–0.65)
Eosinophils	0–0.9 G/l	0.05 (0–0.68)	0.06 (0–2.8)	0.11 (0–0.42)^*^	0.17 (0–1.19)	0.23 (0–0.98)	0.55 (0–2.3)
Basophiles	0–0.1 G/l	0 (0–0)	0 (0–0)	0 (0–0)	0 (0–0.11)	0 (0–0.11)	0 (0–0.12)
Urea nitrogen	≤5.,5 mmol/l	6.25 (2.6–9.8)	4.75 (2.7–6)	4.4 (2.8–7)	4.65 (1.9–6.5)	4.5 (1.4–7.7)	4.65 (3.8–8.2)
Creatinine	≤110 μmol/l	80.83 (59.0–133.8)^*^	68.645 (58.1–119.9)	79.54 (61.6–115.2)^*^	63.605 (50.9–110.6)	71.265 (54.5–97.6)	57.105 (10–96.9)
Total protein	40–80 g/l	75.65 (64.9–91.4)	75.7 (66.9–90.8)	75.95 (67.5–88.1)	74.1 (66–88.5)	74.3 (62.2–89.4)	75.25 (64.5–81.9)
Albumin	≤40 g/l	33.65 (28.9–38.7)^*^	34.15 (21.9–41)^*^	33.2 (28.6–45.1)^*^	32.85 (23.4–40.9)^*^	34.6 (28.2–39.6)^*^	37.3 (29.9–40.4)
Bilirubin	≤8.5 μmol/l	3.885 (1.7–14.3)^*^	2.27 (1.5–5.5)^*^	1.7 (0.7–2.52)^*^	2.04 (0.46–14.6)^*^	1.74 (0.91–5.12)^*^	0.815 (0.11–1.43)
AST	≤80 U/l	74.45 (48.6–121.8)	85.95 (56.5–130.7)	74.85 (62.9–95.2)	76.6 (58–110.7)	76.05 (64.7–108.3)	89.15 (59.2–146.9)
γ-GT	≤36 U/l	16.35 (8.4–23.6)^*^	21 (9.8–32.1)^*^	19.4 (12.8–63.3)^*^	21 (13.1–64.4)	17.65 (10.3–75.2)^*^	38 (29.4–71)
GLDH	≤16 U/l	5.345 (3.3–26.5)^*^	5.6 (2.6–26.3)	5.915 (4.1–13.7)^*^	5.77 (3.5–20.6)^*^	7.43 (1.3–26.9)^*^	15.13 (10.09–47.52)
CK	≤245 U/l	244 (95–824)	142 (70–710)	150.5 (78–387)	159.5 (79–1538)	193 (97–480)	154 (105–795)
P	1.5–3 mmol/l	1.5 (1.1–3.2)	2.1 (1.3–2.7)	2.35 (1.7–2.7)	2.25 (1.6–2.9)	2.2 (1.7–2.8)	2.2 (1.9–2.4)
Mg	0.74–1.44 mmol/l	0.78 (0.73–1.08)^*^	0.9 (0.73–1.01)^*^	0.93 (0.75–1.07)^*^	0.94 (0.72–1.01)	0.98 (0.82–1.06)	1.005 (0.9–1.12)
Ca	2–3 mmol/l	2.045 (0.97–2.34)^*^	2.17 (1.94–2.4)^*^	2.23 (1.92–2.38)^*^	2.245 (2.09–2.4)^*^	2.285 (1.99–2.43)^*^	2.455 (2.1–2.71)
Sodium	130–150 mmol/l	135 (131–139)	137.5 (135–140)	138 (133–143)	138 (134–140)	137.5 (135–140)	140 (131–144)
Potassium	3.5–5.3 mmol/l	4.58 (3.8–5.9)	4.605 (4.0–5.5)^*^	4.845 (4.4–6.1)	5.19 (4.4–5.7)	4.985 (4.4–6.6)	5.27 (4.78–6.01)
Chloride	98–106 mmol/l	98 (90–102)	97 (91–100)	95 (93–99)	96 (92–102)	96 (92–100)	97 (93–104)

*Note*: Values are medians (minimum–maximum)

^*^P < 0.01 (Wilcoxon signed-rank test for paired samples)

### Environmental investigations

In total 310 ticks, three professionally hunted roe deer and one wild boar were examined. Altogether 209 questing ticks (79 adults, 123 nymphs and 7 larvae), 74 engorged ticks from cows and 27 engorged ticks from two hunted roe deer were collected. All were identified as *I. ricinus.* Out of the questing ticks 1.9% (three adults and a pooled sample of 5 nymphs) were PCR-positive for *A. phagocytophilum.* The collected engorged ticks were all adults and 14 out of 74 (18.9%) engorged ticks from cows and 8 out of 27 (29.6%) engorged ticks from two roe deer were PCR-positive for *A. phagocytophilum.* The spleen tissue samples from roe deer were all (3/3) PCR-positive for *A. phagocytophilum,* while the spleen sample from the wild boar was negative.

### Bayticol® treatment

Bayticol® has a withholding period of 8 days for milk in Germany. Consequently the treatment was stopped immediately after calving of the heifers. Seven out of eight treated heifers stayed uninfected with *A. phagocytophilum* during the treatment and only on animal (no. 7) became positive while treated with Bayticol®, 2 weeks after the second treatment. Two animals (nos. 15, 16) did not become positive in the entire period of observation after seven or two rounds of Bayticol® treatment before they calved. The remaining five heifers were either real time PCR-positive for *A. phagocytophilum* in the following three to 18 weeks (nos. 28, 52, 53, 61) or showed antibodies against *A. phagocytophilum* 13 weeks after calving, which was the time when the Bayticol® ended (Fig. 1).

### Gene sequences

The sequences from this study are available in GenBank under the accession numbers KU587048–KU587126. Table 4 shows the nucleotide differences and Table 1 the distribution of the different variants for the four partial gene sequences in the herd. Comparison to the most identical gene sequences in GenBank is provided in Additional file 3: Table S2. Nomenclature of the found variants is not official, but has been previously used by other workers [17, 23, 25].

**Table 4:** Nucleotide differences of *Anaplasma phagocytophilum* in the amplified partial genes

Gene	Variant^e^	Host (*n*)	Nucleotide position												
			76	80	84	175	237								
*16S rRNA* ^a^	16S-7 (I)	tick (1)	G	A	A	A	G								
(497 bp)	16S-19 (V)-like	roe deer (1)	A	G	A	C	A								
	16S-20 (W)	cow (20), ticks (3)	A	A	A	C	G								
	16S-21 (X)	roe deer (2)	G	A	A	C	G								
	16S-22 (Y)	cow (1), ticks (1)	G	A	G	C	G								
			780	840											
*groEL* ^b^	g-15 (N)	cow (3)	A	T											
(530 bp)	g-18 (X)	cow (10)	G	C											
			249	265	291										
*msp2* ^c^	m2-9 (J)	roe deer (1)	T	C	T	further 45 nucleotide differences to m2-26 and m2-27									
(893 bp)	m2-26	cow (9)	T	A	A										
	m2-27	cow (2)	A	C	G										
			375	390	405	411	427	450	462	510	516	603	612	672	678
*msp4* ^d^	m4-13 (*n*)	cow (1), tick (1)	G	G	T	G	A	T	T	C	C	T	C	A	C
(343 bp)	m4-49	cow (12), tick (1)	T	A	C	A	G	T	C	C	T	C	T	A	C
	m4-50	cow (3)	T	A	C	A	G	C	C	T	C	C	T	G	T
	m4-51	cow (5), tick (2)	C	G	T	G	A	T	T	C	T	C	T	A	C

*Note*: *Anaplasma phagocytophilum* HZ complete genome (NC_007797) was used as reference strain analogous to Silaghi et al. [23]

Nucleotide positions indicate the relative position to the genes:

^a^1433 bp of rrsA 16S ribosomal RNA (Gene ID: 3930754)

^b^1653 bp of groEL chaperonin groEL (Gene ID: 3930333)

^c^1098 bp of APH_1361 major surface protein 2 (Gene ID: 3930710)

^d^849 bp of *msp*4 major surface protein 4 (Gene ID: 3930710)

^e^Not official nomenclature; letters in parentheses are based on nomenclature in other publications [17, 23, 25]

### *16S rRNA* gene sequences

The amplification of the *16S rRNA* gene was successful in 29/30 previously selected samples and 5 different gene variants were found. From 21 tested samples of the heifers and the cow two variants here called “16S-20 (W)” (*n* = 20) and “16S-22 (Y)” (*n* = 1) could be observed. The heifer without clinical signs had variant “16S-22 (Y)”. In three roe deer samples two variants here called “16S-21 (X)” (*n* = 2) and “16S-19 (V)-like” (*n* = 1) could be observed. In engorged ticks three variants called “16S-20 (W)” (*n* = 3), “16S-22 (Y)” (*n* = 1) and “16S-7 (I)” (*n* = 1) were found. All 29 tested partial *16S rRNA* gene sequences had 99.2–100% identity to each other.

### *GroEL* gene sequences

Amplicons of the partial *groEL* gene could be obtained in only 13 cow samples and two different variants were observed: variant “g-15 (N)” (*n* = 3) and variant “g-18 (X)” (*n* = 10). The identity between the partial *groEL* gene sequences was 99.6–100% among each other. Interestingly in no. 22 initially variant “g-18 (X)” was detected while 4 month later variant “g-15(N)” was observed in the same animal (Table 1).

### *Msp2* gene sequences

Analysis of the partial *msp2* gene revealed amplicons in 12/30 selected samples which could be analysed. In cow samples two variants “m2-26” (*n* = 9) and “m2-27” (*n* = 2) were detected and in roe deer variant “m2-9 (J)” (*n* = 1). Variant “m2-26” and “m2-27” differ only in three nucleotide positions from each other, whereas “m2-9 (J)” differs in further 45 nucleotide positions to “m2-26” and “m2-27” (Table 4). Therefore the *msp2* gene sequences had an identity of 91.9–100% to each other.

### *Msp4* gene sequences

The amplification of the partial *msp4* gene succeeded in 25 samples and showed four variants: variant “m4-13 (*n*)” (*n* = 2), variant “m4-49” (*n* = 13), variant “m4-50” (*n* = 3) and variant “m4-51” (*n* = 7). All four variants occurred in cattle samples and except for variant “m4-50” also in tick samples. The sequence identity between the partial *msp4* gene sequences was 96.5–100% to each other.

## Discussion

### Clinical picture

Prevalence of *A. phagocytophilum* infections of 27.6% confirmed by real-time PCR is higher in this herd than has been described before for other herds. Other workers found 17.1% positive by nested PCR (12/70 cows and heifers) [6] or 20% positive in a real-time PCR (4/20 cows and heifers) [11]. To our knowledge, a prevalence as high as 78.9% for natural *A. phagocytophilum* infections in naïve heifers confirmed by real-time PCR is described here for the first time. Observed clinical symptoms of TBF in this study (fever, reduced milk production, discharge from eyes and nose, lower limb oedema and stiff walking) match previous clinical case reports [4, 11, 13, 26, 27] as well as observed symptoms after experimental infections [5, 7, 28]. Only heifers that went to the pastures for the first time showed typical clinical symptoms after infection. In contrast to other reports [9, 29, 30] no abortions occurred in this study. Cattle pastured for more than one pasture period are susceptible for re-infections with *A. phagocytophilum*, but show no or very mild clinical symptoms [1, 7]. This is in accordance to our observations with heifers nos. 22, 46, 57, 58 and 61 for which a re- or superinfection was demonstrated by PCR, but no clinical signs were observed (Table 1, Fig. 1). An explanation may be that immunity after an infection with *A. phagocytophilum* can last from 2 weeks up to more than 1 year and that these variants share common antigens [31]. In our study, fever was the first detectable sign of an infection with *A. phagocytophilum*. All real-time PCR-positive heifers except for no. 59 showed typical high fever; therefore measuring body temperature is a suitable initial examination for the detection of infected animals. In contrast, three heifers showed fever in the first week of pasturing without any laboratory evidence for an infection with *A. phagocytophilum.* The fever of no. 57 was very likely metritis-associated whereas the fever of the other two heifers (nos. 14, 59) could not be explained. There are possibly other (pasture associated) infections that might have caused high fever in this herd. The farm is located in the core region where Schmallenberg-Virus (SBV) was first found in late 2011 [32]. To rule out that fever not explained by *A. phagocytophilum* was caused by SBV, all blood samples were additionally screened for SBV by PCR and serology with the outcome that the complete herd seroconverted in weeks 38–40 in 2011 [33]. By that time, only three heifers were still *A. phagocytophilum-*negative. It may be speculated that clinical TBF cases in endemic areas are associated with a pasture management that keeps heifers inside unexposed to ticks until they become cows and then show milk depression and other clinical signs of infection with *A. phagocytophilum*. Grazing of heifers on pastures would to a certain extend lead to natural *A. phagocytophilum* infection without the risk of milk depression, abortion or other clinical signs later in life time of these animals regardless of the particular strain involved in the secondary infection. We cannot rule out that the re-infections are indeed persistent infections, which would make cattle a suitable reservoir host for *A. phagocytophilum*, but we rather consider the infection short-lived. However, the finding that not a single animal was PCR-positive in the herd screening in January 2012 argues against a reservoir role of cattle.

### Cytological, serological, haematological and biochemical examination

Reports of experimental infections of cattle with *A. phagocytophilum* reveal that morulae occur after 5–8 days post infection [5, 7]. Thus, the first infections with *A. phagocytophilum* in this herd took place in the first days of pasturing. Maximum seroprevalence of 36.2% in July 2011 in this herd indicates a high level of endemicity in this area. Other workers describe higher seroprevalences with a maximum of 63% in September in Switzerland [6] or two peaks with a seroprevalence of 75% and 80% in June and November in France [11]. These differences are probably due to variations in vector activity depending on the climate. Prior to pasturing the herd was serologically negative. We assume that the serological situation of the herd must have been similar in the previous year, because of reported typical clinical signs of TBF and the first laboratory evidence for *A. phagocytophilum* in this herd in 2010 [8]. This might be due to a complete fading of antibodies between the pasture seasons and matches the half-live of bovine IgG of 17–22 days [34]. New increases in antibody titers during the pasture season are probably due to re- or superinfections with identical or differing *A. phagocytophilum* variants. The phenomenon of undulating antibody titers during one season was already described by others but the possible involvement of different *A. phagocytophilum* strains was not investigated [7, 31, 35].

Most of the haematological findings match reports from clinical cases [5, 13, 26] as well as cases after experimental infections [4, 7, 28]. The most important haematological finding is severe leucopenia that was also found in the present study. Thrombocytopenia and eosinopenia are often described in *A. phagocytophilum* infections [1], but they were not statistically significant in our results. To our knowledge, there is only one report about biochemical findings in cows after experimental infection with *A. phagocytophilum* [4] and it also shows a significant decrease in creatinine and bilirubin. This is the first report about a statistically significant decrease in albumin, γ-GT, GLDH, magnesium and calcium in naturally infected cattle. Due to the small number of animals in the statistical analysis the validity of these results remains to be proven.

### Environmental investigations

In our study 100% of the roe deer (*n* = 3) were positive for *A. phagocytophilum*. Despite the small number of involved animals, this result matches prevalences of *A. phagocytophilum* found in roe deer in Germany with 94% and 98.9%, respectively [25, 36]. There is only one report about prevalences of *A. phagocytophilum* of wild boars in Germany with a prevalence of 12.5% [37], but other studies from eastern European countries also found quite low prevalences in wild boars between 2.7–12.0% [14]. Thus, the negative result of the wild boar in our study was not surprising. Prevalences of *A. phagocytophilum* in questing ticks from Germany range between 1.0–17.4% [14]. The detected prevalence of 1.7% in questing ticks observed in our study matches the results of other reports. In contrast reports with engorged ticks from roe deer show prevalences of *A. phagocytophilum* DNA of 86.1% (285/331, only adults) and 81% (245/301; adults, larvae and nymphs), respectively [25, 38]. We found a much lower prevalence of 29.6% (8/27) in engorged ticks from roe deer. As the DNA amount in all samples was within one log level we assume the result is not a matter of DNA quality or quantity. Palomar et al. [39] found a prevalence of 30.5% (61/200) in engorged *I. ricinus* from cows and Venclíková et al. [40] found a prevalence of 16.6% (33/199) in engorged *I. ricinus* from sheep. We found a similar prevalence of 18.9% (14/74) in engorged *I. ricinus* from cattle. However, it remains unclear whether engorged ticks were positive for *A. phagocytophilum* before the blood meal or if the positivity is a remnant of the current blood meal.

### Gene sequences

Each of the PCRs used may have a different sensitivity and the amplicon lengths varies from 350 bp to 893 bp. However both facts do not entirely explain why some of the loci could not be amplified (Table 1). Five different partial *16S rRNA* gene variants of *A. phagocytophilum* were detected. The main variant found in cattle was “16S-20 (W)”. This variant was found in all heifers with clinical symptoms and in engorged ticks from cattle, but not in roe deer. This variant has previously been described in infected cattle in Switzerland and Turkey [41, 42], in sheep in Norway [28, 43], in chamois (*Rupicapra rupicapra*), mouflon (*Ovis musimon*) [17, 44] and ticks [45]. There is a repeating occurrence of variant “16S-20 (W)” in infected cattle independently from the geographical origin, therefore this variant seems to be important in causing clinical infections with *A. phagocytophilum* in cattle, at least in Switzerland and Germany.

Variant “16S-22 (Y)” was found in one heifer (no. 59) and in one engorged tick. Interestingly this heifer did not show any clinical signs despite daily monitoring. This variant has previously been described in goats [41], roe deer, mouflon in Austria [17], roe deer, mouflon, and fallow deer in central Germany [46], Swedish moose (*Alces alces*) [47], rodents in Florida [48] and ticks [45, 49].

Variant “16S-21 (X)” has previously been described in goats, roe deer, chamois [17, 41, 44, 50, 51] and ticks [45]. In the USA this variant has often been found in white-tailed deer (*Odocoileus virginianus*) and is identical to the human apathogenic variant Ap-V1 [52].

Variant “16S-7 (I)” has previously been described in roe deer [17, 36] and variant “16S-19 (V-like)” has not been described before. Recent work demonstrated variant X and I in roe deer and mouflon in central Germany [46].

In cattle, we found two different partial *groEL* gene variants. Variant g-18 (X) has been previously found in cattle in France [53], sheep in Norway [28] and ticks in Spain [54] Variant g-15 (N) and the found partial *msp2* gene variants have never been described before. The partial *msp4* gene variant “m4-13 (*n*)” has been found previously in roe deer [17, 53]. Variant “M4-51” has been previously described in cattle in France [53] and ticks in Norway [49]. Variants “M4-49” and “M4-50” have not been described before.

These different variants seem to be present throughout one season as they were found repeatedly indicating a stable circulation of different *A. phagocytophilum* strains in this area at least for one season (Table 1). Only one observed heifer (no. 59) did not show any clinical signs when first infected with *A. phagocytophilum*. It can be ruled out that clinical signs in this heifer have been overlooked, because of the daily measuring of body temperature over the whole pasture period. Interestingly no. 59 was the only heifer with 16S-22 (Y) and M4-13 (*n*) gene variants (Table 1) which might be apathogenic for cattle. This is supported by the fact that this *16S rRNA* gene and *msp4* gene variants were previously found in roe deer in Austria [17]. Roe deer are likely a main reservoir host for *A. phagocytophilum*, but not for cattle-pathogenic variants [2, 55]. Transmission of variants between cattle and roe deer can possibly occur due to a spillover effect [53].

This is the first report about re- and superinfections with various four locus variants of *A. phagocytophilum* in cattle during one grazing period. Animal no. 22 was infected with three different strains with different *msp4* gene variants in May, July and October while no. 57 was infected with *A. phagocytophilum* having the same M4-49 gene variants in June and September 2011 (Table 1). This indicates that infection with *A. phagocytophilum* probably does not result in sterile immunity. Meanwhile many genetic variants have been described based on the genes used here or other genetic markers worldwide [2]. With the various re- and superinfections found, we postulate that this would happen to cattle in any other country where *A. phagocytophilum* is endemic. Although genetic heterogeneity had been described for cattle before [2], the genetic heterogeneity in the cattle samples described here was surprising. This heterogeneity may be even larger, as we investigated only four genetic markers. This is even more notable, because all samples were from a very small geographic region (less than 1 km^2^) during one pasture season and every cow in this study was born on this farm. Red deer is supposed to be a suitable reservoir host of *A. phagocytophilum* for infections of domestic ruminants, but not for humans, horses and dogs [56]. Interestingly, the red deer does not exist in the pasture area; their habitat starts approximately 20 km north from the farm. The pasture areas are regularly frequented by wild boars. Wild boars and hedgehogs were identified to be possible reservoir hosts for *A. phagocytophilum* for humans, horses and dogs and possibly also for sheep and cattle [2]. Single wild boars can cover distances of more than 100 km [57], so they might serve as a “bridging”-host from red deer to cattle in this area.

### Control and prevention

Economic losses due to TBF in lactating cows may best be prevented by allowing first and second season grazing cattle to acquire at least partial immunity in endemic areas. Dry cows should be kept in stables though, as they show more severe clinical symptoms than lactating cows [4]. Furthermore, the risk of abortion due to infection with *A. phagocytophilum* is increased in the last trimester of pregnancy [29].

Avoiding pastures with typical tick habitats, by fencing out certain tick infested areas or by avoiding typical tick population peaks in May–June and September might be an option for those farms that cannot bring their heifers to pastures. However, even though this might reduce the tick burden, but will not protect completely from infections with tick-borne diseases.

Bayticol® is the only authorized repellent for cattle with long acting effect (3 weeks) against ticks on the German market. The results of the farmer’s treatment indicate a relatively good effectiveness of flumethrin, but the number of treated animals in this study was too low to make a statement about the overall effectiveness of the drug. Stuen et al. [58] showed that Bayticol® could reduce the tick burden in sheep but could not protect the animals from seroconversion. Bayticol® has 8 days of withholding period on milk; therefore, it is not suitable for dairy herds.

## Conclusion

In the observed dairy cattle herd we found a high prevalence of *A. phagocytophilum*-infections and clinical signs allowing a tentative diagnosis of TBF. Four locus sequence typing (*16S rRNA*, *groEL*, *msp2* and *msp4*) showed that several genetic variants sympatrically circulate in this small geographical region. Cows harbored other genetic variants than roe deer. This might be indicative for either distinct transmission cycles or host selection/restriction of particular *A. phagocytophilum* variants. Pathogenicity, host tropism and possible reservoir hosts of the detected genetic variants remain unclear and need to be further investigated.

## Additional files

Additional file 1: Table S1. Primers used for the PCR amplifications and sequence analysis of *Anaplasma phagocytophilum* in this study. (DOC 33 kb)

Additional file 2: Figure S1. Overview of the herd seroprevalence during one season (based on the data shown in Table 2). First samples were taken in May shortly before the animals went to pasture. Despite two animals with a titre of 1:50 (dark asterisk), no measurable antibody titres were detected. This changed over the following months (x- axis). The left y-axis shows the percentage of seropositive animals (blue, cows; green, heifers). Logarithmic titres are given on the right y-axis. Boxplots in gray display the median titre (black bars), upper and lower quartile and the upper wisker. Outliers (asterisks) were found in May and January. In January only two heifers had antibody titres of 1:100 and 1:200, while 10 further animals had a remaining antibody titre of 1:50 (darker asterisk). (TIFF 54 kb)

Additional file 3: Table S2. Accession numbers of gene variants of *A. phagocytophilum* in comparison with variants from GenBank. (DOC 42 kb)

## Abbreviations

*16S rRNA*: 16 Svedberg ribosomal ribonucleic acid
GLDH: Glutamate dehydrogenase
*groEL*: Heat shock protein HSP60, also known as chaperonin 60
HGA: Human granulocytic anaplasmosis
IFAT: Indirect immunofluorescence assay
*msp2*: Major surface protein 2
*msp4*: Major surface protein 4
SBV: Schmallenberg virus
SD: Standard diviation
TBF: Tick-borne fever
γ-GT: γ-glutamyltransferase
