# Supplementary material for: Epidemiology, genetic variants and clinical course of natural infections with Anaplasma phagocytophilum in a dairy cattle herd
Source: Parasit Vectors. 2018 Jan 8;11:20. doi: 10.1186/s13071-017-2570-1 (PMC5759301; doi:10.1186/s13071-017-2570-1)
Supplement: Supplementary file 1 — Primers used for the PCR amplifications and sequence analysis of Anaplasma phagocytophilum in this study. (DOC 33 kb) [file 13071_2017_2570_MOESM1_ESM.doc]

**Additional file 1: Table S1 Primers used for the PCR amplifications and sequence analysis of *Anaplasma phagocytophilum* in this study**

| **Target Gene** | **Primers 5’-3’ ^a^** | **Cycle conditions ^c^** | **Reference** |
| --- | --- | --- | --- |
| *16S rRNA* | First reaction:  ge3a: CACATGCAAGTCGAACGGATTATTC  ge10r: TTCCGTTAAGAAGGATCTAATCTCC  Nested reaction ^b^:  ge9f: AACGGATTATTCTTTATAGCTTGCT  ge2: GGCAGTATTAAAAGCAGCTCCAGG | 40 cycles:  30 sec 94 °C, 30 sec 55 °C, 1 min 72 °C  25 cycles:  25 sec 94 °C, 30 sec 60°C, 25 sec 72°C | [18] |
| *groEL* | First reaction:  EphplgroEL-F: ATGGTATGCAGTTTGATCGC  EphplgroEL-R: TCTACTCTGTCTTTGCGTTC  Nested reaction ^b^:  EphplgroEL-F: ATGGTATGCAGTTTGATCGC  EphgroEL-R: TTGAGTACAGCAACACCACCGGAA | 40 cycles:  30 sec 94 °C, 30 sec 55 °C, 45 sec 72 °C  40 cycles:  30 sec 94 °C, 30 sec 55 °C, 45 sec 72 °C | [19] |
| *msp2* | msp25: TTATGATTAGGCCTTTGGGCATG ^b^  msp23: TCAGAAAGATACACGTGCGCCC ^b^ | 35 cycles:  1 min 95 °C, 1 min 62 °C, 1.5 min 72°C | [20] |
| *msp4* | First reaction:  MSP4AP5: ATGAATTACAGAGAATTGCTTGTAGG  MSP4AP3: TTAATTGAAAGCAAATCTTGCTCCTATG  Nested reaction ^b^:  msp4f: CTATTGGYGGNGCYAGAGT  msp4r: GTTCATCGAAAATTCCGTGGTA | 40 cycles:  30 sec 94 °C, 45 sec 54 °C, 1 min 72 °C  40 cycles:  30 sec 94 °C, 45 sec 54 °C, 1 min 72 °C | [22]  [21] |

^a^ Please note that the primer sequences were taken from the cited literature, but cycling conditions might have been modified according to the chemistry used in our experiments.

^b^ Primers used for the sequencing reactions

^c^ all PCR reactions: 15 min 95°C initial activation; 7 min 72 °C final extension
