# Supplementary material for: Epidemiology, genetic variants and clinical course of natural infections with Anaplasma phagocytophilum in a dairy cattle herd
Source: Parasit Vectors. 2018 Jan 8;11:20. doi: 10.1186/s13071-017-2570-1 (PMC5759301; doi:10.1186/s13071-017-2570-1)
Supplement: Supplementary file 3 — Accession numbers of gene variants of A. phagocytophilum in comparison with variants from GenBank. (DOC 42 kb) [file 13071_2017_2570_MOESM3_ESM.doc]

**Additional file 2: Table S2** Accession numbers of gene variants of *A. phagocytophilum* in comparison with variants from GenBank

| Gene | variant^e^ | host | Our accession numbers | Percent identity with |
| --- | --- | --- | --- | --- |
| *16S rRNA* | 16S-7 (I) | tick | KU587119 | 100% identical to Gen Bank GU236534, *Capreolus capreolus*, Slovenia |
| (497bp) | 16S-19 (V)- like | roe deer | KU587126 | 99% identical to Gen Bank KM215231, *Ixodes ricinus*, Slovenia |
|  | 16S-20 (W) | cow, ticks | KU587098 – KU587115,  KU587117, KU587118, KU587120 – KU587122 | 100% identical to Gen Bank CP015376, sheep, Norway |
|  | 16S-21 (X) | roe deer | KU587124, KU587125 | 100% identical to Gen Bank AF012528, *Ixodes ricinus*, France |
|  | 16S-22 (Y) | cow, ticks | KU587116, KU587123 | 100% identical to Gen Bank KP245908, *Ixodes ricinus*, Poland |
| *groEL*  (530bp) | g-15 (N) | cow | KU587051, KU587052,  KU587056 | 99% identical to Gen Bank KJ832483, cattle, France |
|  | g-18 (X) | cow | KU587048 – KU587050,  KU587053 – KU587055,  KU587057 – KU587060 | 100% identical to Gen Bank KJ832487, cattle, France |
| *msp2* | m2-9 (J) | roe deer | KU587072 | 99% identical to Gen Bank JN244019, *Capreolus capreolus*, Germany |
| (893bp) | m2-26 | cow | KU587061 – KU587066,  KU587068 – KU587070 | 98% identical to Gen Bank CP006618, dog, USA |
|  | m2-27 | cow | KU587067, KU587071 | 98% identical to Gen Bank CP006618, dog, USA |
| *msp4* | m4-13 (*n*) | cow, tick | KU587091, KU587097 | 100% identical to Gen Bank KJ832653, *Capreolus capreolus*, France |
| (343bp) | m4-49 | cow, tick | KU587073 – KU587075,  KU587080 – KU587083,  KU587087 – KU587090,  KU587092, KU587095 | 99% identical to Gen Bank KJ832672, cattle, France |
|  | m4-50 | cow | KU587076, KU587079,  KU587084, | 99% identical to Gen Bank KJ832668, cattle, France |
|  | m4-51 | cow, tick | KU587077, KU587078,  KU587085, KU587086,  KU587093, KU587094,  KU587096 | 100% identical to Gen Bank KJ832659, cattle, France |
